# Supplementary material for: Victimizations and surviving of workplace violence against waitresses in southern Ethiopia
Source: PLoS One. 2021 Dec 31;16(12):e0261883. doi: 10.1371/journal.pone.0261883 (PMC8719772; doi:10.1371/journal.pone.0261883)
Supplement: S1 File — (DOCX) [file pone.0261883.s001.docx]

የተጠያቂዋ መሠረታዊመረጃዎች፡

1. ዕድሜ?

2. የጋብቻ ሁኔታ?

3. የትምህርት ሁኔታ?

4. በስራው ውስጥ የቆየችበት አመት?

ጥያቄዎች

1. አሁን በምትሰሪበት የስራ ቦታ፡ ፆታን መሠረት ያደረገ ጥቃት (በሴትነትሽ ወይም እዚህ ቦታ ላይ በመስራትሽ ምክንያ) ምን ያክል ያጋጥማል?
   1. አሁን ወይም ከዚህ በፊት በተመሳሳይ የስራ ዘርፍ ውስጥ በተሰማራሽባቸው ጊዜያት፡ ፆታን መሠረት ያደረገ ጥቃት (በሴትነትሽ ወይም እዚህ ቦታ ላይ በመስራትሽ ምክንያ) አጋጥሞሽ ያውቃል?
   2. አጋጥሞሽ ከሆነ፡ ምን አይነት ጥቃት (አካላዊ፣ እንደ ስድብ ያሉ፡ ሞራል የሚነኩ ንግግሮች፣ የመሳሰሉት) ነው ደርሶብሽ የሚያውቀው?
   3. …..በማን፣ እንዴት፣ መች፣ እና በምን አይነት ሁኔታ ጥቃቱ እንደደረሰብሽ በዝርዝር ብትነግሪኝ?
   4. የደረሱብሽ፡ ይቃቶች ከማንነትሽ (በፆታ፣ በብሄር፣ በሃይማኖት፣ ወይም በኑሮ ሁኔታሽ) ጋር ተያያዥነት ይኖራቸው ይሆን?
   5. ምናልባት በማንነትሽ (በፆታ፣ በብሄር፣ በሃይማኖት፣ ወይም በኑሮ ሁኔታሽ) ምክንያት ተጨማሪ ጥቃቶች ወይም በስራ ቦታሽ መድሎዎች ደርሰውብሽ የሚያውቁ ከሆነ፡ ብትነግሪኝ?
2. በስራ ቦታሽ ለሚደርሱብሽ ማናቸውም ጥቃቶች፡ በምን መልኩ ነው ምላሽ የምትሰጪው?
   1. በስራ ቦታሽ ላይ ማናቸውም አይነት ጥቃቶች ሲደርሱብሽ፡ ምን ታደርጊ ነበር ወይም ታደርጊያለሽ?
   2. ለሚመለከታቸው አካላት፡ ማለትም ለፖሊስ፣ ለስራ አለቆችሽ፣ ለጥበቃዎች፣ ወይም ለባለቤቶቹ አመልክተሸ ታውቂያለሽ?
   3. …..ለምንድነው እንደዚያ ያደረግሽው….ወይም ያላደረግሽው?
   4. አመልክተሸ ወይም አቤቱታ አቅርበሽ የምታውቂ ከሆነ፡ የተሰጠሸ ምላሽ ምን ይመስል ነበር?
   5. መሰል የስራ ቦታ ላይ ጥቃቶች ሲደርሱብሽ፡ በብዛት ወይም ዘውትር የምታደርጊውን ነገር ብትነግሪኝ?
3. በስራ ቦታሽ ላይ ባጋጠመሽ (ደርሶባት የሚያውቅ ከሆነ) ጥቃት ምክንያት የደረሰብሽ አካላዊ፣ ማህበራዊ፣ ስነ-ልቦናዊ ወይም ሌላ ችግር ካለ ብትነግሪኝ?

……ከማህበራዊ ህይወት መገለል፣ አካላዊ ጉዳት፣ ህመም፣ ለተጨማሪ ጥቃቶች የመጋለጥ ፍርሃት፣ ድብርት፣ ጭንቀት፣ የብቸኝነት ስሜት፣ የተስፋ-ቢስነት ስሜት…ወዘተ?

1. ወደስራው ከመግባትሽ በፊት፡ ስለስራ ቦታው ሰላማዊነት የነበረሽ ግምት ወይም እይታ ምን ይመስል ነበር?
   1. እዚህ ስራ ውስጥ ከመቀጠርሽ በፊት በነበሩት ጊዜያት፡ ወደዚህ ስራ ስትገቢ የስራ ቦታ ላይ ጥቃቶች ሊደርስብሽ እንደሞችል ጠብቀሽ ነበር?
   2. መልስሽ አዎ ከሆነ፡ ሊደርሱብሽ የሚችሉትን ጥቃቶች ለመቀበል እራስሽን አሳምነሽ ነው የገባሽበት?
   3. ጥቃቶች ሲደርሱብሽ፡ ለመቋቋም የሚያስችልሽን ምን አይነት ዘዴዎችን ነበር ያቀድሽው?

In-depth Interview guiding questions

Note: please specify whether the violence that interviewees faced is from the side of customer/client, employer/owner, co-worker, boss (abuse of power), owner’s family/relative, stranger, etc

█ substantiate the research by collecting data, asking interviewees if they are victims of violence on accounts of ethnicity, religion, etc

Some types of gender-based violence-for use by the interviewer

❶ verbal violence such as insulting or saying something immoral or humiliating, teasing, etc, bulldozing/scaremongering, intimidating,

❷physical violence, including beatings, slapping/touching female’s sexual/private parts such as buttock, breast, etc, spitting, twitting a hand, slapping a face, rape, attempted rape, simple & aggravated assault

❸Not paying money after use

❹Denying salary--- from the side of employers

1. How prevalent is gender-based violence in your work place?
   1. Have you ever faced gender-based violence now or before while working in similar work places?
   2. Would you please tell me some of the violence types that you faced?
   3. ………..*also ask her to specify where, when (what time), by whom ( whether she has faced it from the side of customer/client, employer/owner, owner’s family/relative, stranger, etc)*
   4. *……also specify if she faced the violence on account of her gender, ethnicity, religion, social class, or any other stratifying variable?*
   5. Have you ever faced violence or discrimination on the basis of your ethnicity, religious affiliation, or belongingness to a social group that is perceived to as a different/minority in the area?
2. How did you react or respond to the violence you have faced?
   1. What did you do after facing the violence or discrimination in your work place?
   2. Have you ever reported to the police, managers/supervisors, [body] guards, the owner, etc……or you just kept silent?
   3. ……….and why did you do that ……or not did so?
   4. If you have ever reported, then how did they/it respond to your complaints?
   5. …………..What is the most common thing you do whenever you face such violence?
3. What physical, psychological, and social challenges have you faced because of the violence?

……… *social exclusion, physical damage, illness or sickness, fear or feelings of intimidation, depression, anxiety, loneliness, feelings of helplessness, feelings of worthlessness, etc*

1. What was your expectation regarding the possibility of being exposed to gender-based violence in such work places?
   1. Have you ever expected or were you expecting to be exposed for such workplace violence when you decide to work here?
   2. ….if yes, then, have you convinced yourself to accept victimizations?
   3. …….what strategies have you planned to cope-up with the situation?

| No (code) | Variable | Categories | Frequency |
| --- | --- | --- | --- |
|  | Age |  |  |
|  | Marital status |  |  |
|  | Education |  |  |
|  | Years in the work |  |  |
