# Supplementary material for: Victimizations and surviving of workplace violence against waitresses in southern Ethiopia
Source: PLoS One. 2021 Dec 31;16(12):e0261883. doi: 10.1371/journal.pone.0261883 (PMC8719772; doi:10.1371/journal.pone.0261883)
Supplement: Attachment — Submitted filename: PONE-D-21-09783_Reviewers Edit.docx [file pone.0261883.s002.docx]

PLOS ONE

**Victimizations and Surviving of Workplace Violence against Waitresses in Southern Ethiopia**

--Manuscript Draft--

# Victimizations and Surviving of Workplace Violence against Waitresses in Southern Ethiopia

Abstract

**Background**: Women are obliged to take on vulnerable forms of employment that fail to protect their basic labor rights. Exposure to workplace violence is especially high for those women who work within the agriculture, the hotels, restaurants and catering, the transport as well as the manufacturing sectors. In this context, we explored workplace violence against women working as waitresses in various hotels, restaurants, cafeterias and grocery stores of Wolaita Sodo town, southern Ethiopia.

**Methods**: Cross-sectional study was undertaken with descriptive and exploratory study designs. Qualitative data were collected by using in-depth interview method in which 16 waitresses were interviewed. Data were voice recorded, transcribed, and analyzed searching themes and patterns in the data.

**Result**: While we found high incidence of workplace violence against the waitresses, the level of exposure to the violence varies across various circumstances, including working in large and small towns, the situation of the owners/supervisors, public’s insight of the position, waitresses’ ability to speak the local language, the role of the waitresses, customers’ behavior and the specific context in which waitresses work. Waitresses are generally exposed to all forms of violence including spitting, throwing objects, simple physical assault, touching on private parts, and intimidation, among which verbal abuse and emotional exploitations are found to be the most common. Furthermore, the results revealed that though waitresses rarely experience violence from female customers, the most common perpetrators involved in the practice were males. Amid widespread exposure of waitresses to all forms of workplace violence, ignorance, mostly motivated by fear of losing one’s job, has been the common way waitresses respond to the violence.

**Conclusion:** The study implies the vulnerability state of waitresses partly due to lack of adequate awareness regarding the rights and obligations expected of an employee when working in such organizations. This is again exacerbated by the lack of formal employment procedures in such organizations. Therefore, awareness creation and supervisory activities are expected from the media, workers’ and social security office of the government, police and other concerned bodies.

Key words: Exposure, Coping strategies, Waitress, Workplace violence, Reaction

## Introduction

In the past, the economic and social status of women was closely linked to that of their husbands but currently, there has been a huge improvement in the economic, political, and social status of women, of which employment is an integral part (Banerjee, 2009). Women are not just mothers but are also workers who represent over 42% of the global paid working population (WHO, 2004a). The number of women joining the workforce has steadily increased all over the world during the last few decades (WHO, 2001). As women move beyond their traditional occupations, they meet new health hazards which may either replace or add to their existing occupational exposure (Kane, nd). Women’s participation in the labor force shows the importance and contribution of women to economic productivity, hence, the need for occupational health and safety concerns (Lu, 2011). However, the beneficial effects of employment on the lives of women are contingent on several factors such as the nature of work, the work environment, income earned and time involved, as well as the social and cultural norms that either fight or approve of women’s work outside the home (Amoako, 2019).

Occupational health is defined as the promotion and maintenance of the highest degree of physical, mental and social well-being of workers in all occupations (Koh & Jeyaratnam, 2001). It comprises all areas of health and safety in the workplace, with a strong emphasis on the primary prevention of hazards (WHO, 2018b). There is a continuous two-way interaction between a person and the physical and psychological working environment: the work environment may influence the person’s health either positively or negatively and productivity is, in turn, influenced by the worker’s state of physical and mental wellbeing (WHO, 2001). According to Tawiah & Mensah (2016), occupational health and safety has significant impact on affective, normative, and continuance commitment of workers.

Addressing the occupational violence which all workers are exposed to is important, but it must not be done at the expense of making visible the gendered and discriminatory dimensions of workplace violence (Lippel, 2018). The issue of occupational safety and health for women is central to an understanding of the working environment. Gender inequalities in the workplace and work-life balance issues have become increasingly important as employment rates of women have continued to grow (EU-OSHA, 2013). Although men tend to be at greater risk of direct physical assault because they are more likely to be found in jobs such as security and the prison service, women are also found in many of the occupations with a high-risk of sexual harassment, violence and threats of violence, working in contact with the public in banks, bookmakers, shops and in solitary settings (TUC, 2017; EU-OSHA, 2014; Lu, 2011). According to Gatimu (2015), violence against women and a minor is a means of control and oppression by the powerful members of the society. Above all, workplace violence can be considered as a reflection of the general and increasing phenomenon of violence in many areas of social life which has to be broadly viewed at the level of the whole society (Ali & Ali, 2014).

Violence against women is the most widespread form of abuse worldwide, affecting one third of all women in their lifetime (Action aid, 2012). Gender-based violence ranges from sexual harassment to rape, domestic violence to trafficking (Pedro, 2013). It is globally recognized as a health, economic development, and human rights concern that both reflects and reinforces inequalities between women and men (Amnesty International, 2004; ILO, 2011). A study undertaken in Zimbabwe, for instance, uncovered the impact of gender-based violence on the reproductive health of women (Mukanangana, Moyo, Zvoushe, et al., 2014). In addition, the negative impacts of gender discrimination on development and poverty alleviation has been found by Alam (2010). While factors such as working alone, working with the public, and in educational settings have been identified as task-related risk factors, the working environment such as organizational setting and managerial style, as well as the workplace culture and external environment are workplace risk factors for the prevalence of gender-based violence against women (ILO, 2011). Canadian Center for Occupational Health and Safety (2021) maintain that working with customers or the public is among the work factors that put people at increased risk of workplace violence and sellers of alcohol are among the occupational groups that tend to be at risk from workplace violence.

Women are at particular risk of violence, harassment and bullying both in and outside the workplace (TUC, 2017; ILO, 2010). It is important to remember that women still typically have the dual burden of household work and caring responsibilities which can and do expose them to the same hazards at home they experience at work, increasing the likelihood of injury (TUC, 2017). The question of women’s equality and security in the workplace is at the nexus of many social issues and social conceptualizations of women: women as workers, as mothers, as victims of violence against women, and sexual and gender-based violence (Coyle, Shrestha, Jung, 2014).

Ethiopia has one of the highest prevalence of sexual, emotional, and physical violence against women in the work place (Yemane, 2004). Gender based violence continues to be a significant and serious human rights and public health issue in Ethiopia (Tsegahun, 2008). Women in Ethiopia are acutely affected by discriminatory policies and gender norms. They are nearly three times as likely as men to be unemployed. Women are obliged to take on vulnerable forms of employment that fail to protect their basic labor rights (UNDP, 2018). Above all, differences between women and men’s exposure to the risk of specific forms of workplace violence are reinforced by sex-segregated workplaces–both horizontally and vertically (ILO, 2011).

According to EU-OSHA (2013), the occupational health risks of female workers tend to focus on their exposure to material, physical and ergonomic hazards, as well as intimidation and discrimination at work. These types of exposures are especially high for those women who work within the agriculture, the hotels, restaurants and catering, the transport as well as the manufacturing sectors. In Ethiopia, there is a high level of workplace injuries that often leads to an extended loss of productive working days. Occupational safety and health services were found to be inadequately organized (Abera, Tadesse, Kiros, et al., 2016). The legal codes in Ethiopia are used to support male superiority imposing lesser stringent sanctions on perpetrators of violence against women whether that happens within or outside wed-lock. Lack of information

about women’s rights, limited access to legal services, insensitivity of law enforcement bodies, and the generally poor status of women in the society are some of the main reasons that accounted for the continuation of violence against women in Ethiopia (Yemane, 2004).

The existence of relevant national legislative frameworks on gender-based violence as well as a workplace-level policy and implementation strategy are also important contextual factors (ILO, 2011). Concerns of workers’ occupational health and safety have been included in Proclamation No. 1/1995 of the constitution of the Federal Democratic Republic of Ethiopia. Article 42 (2) provides that workers have the right to reasonable limitation of working hours, to rest, leisure, to periodic leaves with pay, to remuneration for public holidays as well as healthy and safe work environment. Moreover, Article 35 (8) states that women shall enjoy equal right with men in employment, promotion pay and the transfer of pension entitlement. Above all, article 92 of the Ethiopian Labor Proclamation No. 377/06 clearly spells out the fundamental obligations of an employer with regard to putting in place of all the necessary measures in order to ensure that work places are safe, healthy and free of any danger to the wellbeing of workers. According to Dawit (2006), violence and sexual harassment at workplace is a prohibited action and its transgression will be one of the reasons that are considered for automatic termination of contract of employment for a worker, a process which should ends up compensating the employee and getting the employer punished.

Findings of previous empirical literatures show exposure of employed women to various occupational hazards and ill health conditions, including the impacts of such exposure on women’s overall physical, biological, psychological, and social life. For instance, a study of women’s health working in the mining sector by Botha (2015) revealed that women are still exploited and sexually harassed in the mining industry. Incidents taking place daily vary from whistling; name calling; use of vulgar or derogatory language; display of body parts; physical contact, ranging from touching to sexual assault and rape; to the exchange of sexual favors for promotion. Moreover, Tawiah & Mensah, Ray, et al. (2015) found high level of morbidity in the form of headache, body ache, problems with vision, cough and breathlessness were observed in Brick Kiln and Construction industries of India. A widespread prevalence of sexual harassment among women working in the health care has also been noted (e.g, Subedi, Hamal, & Hari, 2013; Rajbhandari, Subedi, & Kaphle. 2015). Furthermore, the findings of a research into women’s occupational health and safety in the informal economy suggested that maternal market traders face some occupational health risks that have a significant toll on their physical, mental and social health (Amoako, 2019). Moreover, a study of workplace violence among male and female Turkish employees of various sectors undertaken by Akinci and Bilgel (2011) found that nearly half of the workers reported that they had been subjected to a violent event at their workplaces. Workplace violence against women working in the hotel sector, however, is little studied. According to Lippel (2018), workplace sexual violence, and sexual harassment, have received attention in the literature and in regulatory strategies, but other types of violence to which women are disproportionally exposed either at work, because of the nature of their work, or while they travel to and from work, are less often addressed through a

gender lens. The characteristics of violence commonly committed against women differ in critical respects from violence commonly committed against men though both can be victims as well as perpetrators of violence (Morrison, Ellsberg, Bott, 2004).

The objective of the present study was, therefore, to explore workplace violence against women working as waitresses in various hotels, restaurants, cafeterias and grocery stores of Wolaita Sodo town, southern Ethiopia.

# Materials and Methods

## Research design

Cross-sectional study was undertaken with descriptive and exploratory study designs. Using qualitative approach, data were collected from a sample of women working as waitresses pertaining to exposure to workplace violence.

## Selection of Research participants

The participants of the study are women who work as waitresses in different hotels, grocery stores, cafeterias and restaurants of Woliata sodo town, southern Ethiopia. With the aim of analyzing the differential level of exposure to workplace violence within the group, we have purposefully selected equal number of participants representing the group of waitresses working in the restaurant, cafeteria, hotel, and grocery store. Because the process of employment is mostly undertaken in the informal networks and the target groups are not beneficiaries of the formal social security schemes of the local government, the total number of waitresses working in the area was unknown. Consequently, we recruited ten potential participants each from hotels, restaurants, cafeterias, and grocery stores in the first stage of the sampling process. Both the recruitment of the research participants and the selection of the specific workplaces were made on the basis of random sampling technique. Lately, while twelve waitresses have withdrawn from participation in the meantime of data collection, data saturation was attained after sixteen waitresses were interviewed. Accordingly, the final sample size participated in the research was

16. Sex and willingness to participate in the study were the major inclusion criteria we used during recruitment. In addition, waitresses who have only recently employed (within in a period of less than a month) were not included in the study.

## Method and procedure of data collection

Qualitative data were collected using in-depth interview method. An interview checklist, containing unstructured questions was prepared to guide the process of conducting the interview. The questions were prepared in the way they can address the specific research objectives that the research intend to achieve, including: 1) how prevalent is workplace violence against the waitresses and its level of exposure, 2) the types of workplace violence to which waitresses are most commonly exposed, 3) the age-sex and other characteristics of the perpetrators that commonly inflict workplace violence, and 4) the common ways of responding/reacting to

workplace violence. During field visits, we have first contacted the owners/managers of the randomly selected organizations to get access to the waitresses. Once access was gained, interviewees were informed ahead about the purpose and rationale of undertaking the research and provided their oral consent. Then, they were asked the time and place convenient to them to conduct the interview. Data collectors approached the interviewees in a friendly manner and created a rapport which helped to maintain trust. Research participants were asked unstructured questions, followed by probing in the middle of the interview. Both field notes and voice recordings were used in the process.

## Method of data analysis

Data collected using field notes and voice recordings in the local language were first transcribed and then translated to English. Then, data were organized according to the identified themes. The four major themes identified in the data were 1) exposure to workplace violence, 2) underlying factors contributing to differential exposure to workplace violence within the group, 3) age-sex and related socio-demographic characteristics of perpetrators, and 4) the coping strategies against workplace violence. Data interpretation and analysis was undertaken according to the specific research objectives.

# Results

## Victimizations and Incidents of workplace violence

Data have shown that waitresses are highly exposed to workplace violence of different forms. The most reported type of workplace violence was verbal abuse, mostly manifested in insisting the victims to provide their phone number to the male customers as an after-service offer. Whereas compliance on the side of the waitress to the telephone number request usually end up with no further violence, failure to positively reply have been reported to cause subsequent forms of verbal and physical violence. In this regard, insulting with derogatory words and humiliation, bulldozing/scaremongering and intimidation are found to be the common ones. For instance, an interviewee disclosed: “*The most remarkable case I ever experienced was the one I faced in the third month after I joined the work. It happened with a customer who used to come to the restaurant so often and every time he (the customer) comes, he always asks me to give him my phone number. One day, I served him the food he ordered and when he finished eating, he asked me if I could go out with him or give him my phone number. Following my refusal, he warned me that he will not be paying the bill if I continued refusing.”* And another research participant added: *“I have done as a waitress in many places and I have witnessed so many customers who drink a lot and disturb. Most of them ask me to give them my phone number and to spend the night with them. Sometimes this job is assumed as prostitution.”*

In addition to the verbal abuse, incidents of physical violence have also been reported where slapping on the face, beatings, touching on sexual or private parts, spitting, twitting a hand, and other simple physical assaults are commonly experienced by the waitresses. Furthermore, while the victimization of some waitresses is limited to only one or other forms of verbal or physical violence, the case among most interviewees reveals that they are concurrently exposed to both verbal as well as physical violence where the former is followed by the later. Genet’s experience would illustrate the scenario:

*“Once upon a time I was working in a grocery store at a small town called Yabelo. On one occasion, two persons came to my workplace, they were too drunk and asked me to spend the night with them but I kept silent. Mea*nwhile, *they started to i*nsult me; *bu*t *I still kept silent*. *They then began* to *touch my buttock a*nd *b*reast*. I patiently replied to them that my job is waitress, if they want a woman to sleep with; they can find prostitutes outside. Then they said to me, ‘Who are you?’ Finally, they started going out without paying the bill; the owner asked me if I took the money. I then replied to him that I didn’t and I went to them (customers) to ask. One of them slapped my face and tore my clothes into two pieces; windows of the house were broken during quarrels. Finally, police officers arrived and arrested the man."*

## Dynamics of workplace violence

While we found high incidences of workplace violence against women in the study area, research participants also disclosed that the level of exposure to the violence varies across the following circumstances.

**Working in Large and small town*s***: according to the interviewees, incidences of workplace violence against the waitresses tend to increase as one move from larger towns (capital cities) to the smaller ones: “…..*it varies according to the town where I work. For example, when I worked in Addis Ababa, customers have a lot of respect for the waitresses, but it was very different outside of Addis; there were times when they even think about body posture, customers need a waitress with big thick buttocks. They don't want the service or came here just to eat or drink something. They think they came here to eat good food with “good looking girl”. They even prefer between waitresses. They say, ‘I don't want you!’, even if I am assigned to serve of their sit.”* Research participants reported to have worked in various towns of Ethiopia hold the view that customers found in the relatively larger towns and regional or capital cities are better educated and hence respectful of the waitresses.

**The situation of the owners/managers**: data reveal that the incidences of workplace violence against waitresses differ on the basis of the situation of owners/supervisors. While some owners/supervisors attempt to be fair and stand by the side of the waitresses, they commonly prefer to act impartially and help the waitresses during upheavals: *“The supervisor is always by our side. Whenever some customers try to abuse us, she will always tell them that, ‘the*

*waitresses are here to serve you, so you shouldn’t talk to them like that, and you can leave if you don't want’. As a result, If it is not a customer that comes to our hotel very rarely, such as a* b*y passer, all customers commonly served here do not dare to insult us, they respect us.”*

Conversely, there are supervisors/owners of the organizations that act in favor of the abusive customers. According to the interviewees, such owners or supervisors perceive that ‘a customer is a king whatever way he behaves and no matter he abuses the waitresses.’ The narrative of Lemlem stipulates that some owners are not responsive to workplace violence against waitresses: *“….when things get worse, I often report to the owner. But, my pain doesn’t give him sense; what he needs is to maintain the safety of the customers at the expense of ours and keep customers coming to the cafeteria. Though I report what happens with my tears flowing, he never felt my pain. Instead, he gives priority to the customers, not to the waitresses. After all, there is no solution from the owners.”*

Furthermore, the absence of sympathy among most owners and supervisors is accompanied by the lack of self-esteem and the presence of high job-insecurity of the waitresses all of which have the effect of exacerbating workplace violence against the waitresses. For instance, the respo*nse* that: “*It is impossible to judge the owner; after all, it is business for him and I am a waitress here. If I quit, another waitress will come. But, if a customer quits, the owner will lose a lot. Whatever happens, due attention is given to the concerns of the customers, not the waitresses”* would illustrate the fact that some waitresses are skeptical of losing their job and willingly opt to accept the abusive conditions.

**Public’s insight of the position**: It is found that the understanding to the position of waitress and the value placed to the women assuming the status widely varies across customers. And this is related to the manner in which customers and waitresses interact in the workplace. According to the interviewees, the job of waitress is a less prestigious position and “*the assumption held among some customers is that waitresses are part-time prostitutes.”* Interviewees believe that one possible source of such perceptions is the dressing norms to which all waitresses must conform which purposively leave some parts of women’s body naked in a way it seduces and retains male customers. In addition, it is also disclosed that some customers believe waitresses have the responsibility of satisfying the [sexual] demands of the customers: *“Some customers have a distorted view of the job. For example, if you refuse to go out with them, they threaten you saying that they will report to the owner that ‘this girl is not comfortable with us’ so that the owner fires you away.”*

**Language:** The level of exposure to workplace violence has been found to vary on the basis of waitresses’ ability to speak the local language. While most of the research participants, especially the ones working in big hotels, disclosed that they came from another area in search of job opportunities, they unveiled that their exposure to workplace violence is partly related to their inability of speaking the local language (*wolaitatto*). It is also found that waitresses face various sorts of workplace violence on accounts of their ethnicity too: *“Of course, I was attacked not only because of my sex but also because of my ethnicity. When some customers ask me about my ethnicity, I feel discontented. If I disclose to them that I don't speak the language, they tend to*

*question my presence in the area. Some perceive that if I am not able to speak their language, I will not be able to handle them well.”* Moreover, the implication of inability to listen and speak the local language on the emotional/psychological wellbeing of waitresses has also been noted in the present study. Data revealed experiences of feelings of loneliness, sense of being marginalized and excluded, and lack of security among waitresses that do not speak the local language.

**The role of the waitresses**: data reveal that waitresses often contribute to workplace violence sometimes by initiating discontent among customers. Failure to serve the right order, errors of sequence committed when a customer who came first is not able to be served first, and extended delay both to receive orders and to bring what is ordered have been found to be the ways by which waitresses play the role of initiating violence. According to the research participants, while some customers politely call and complain to the waitress in charge of their sit, others do not give them a chance to correct their mistakes. The following account of Kidist clearly depicts the scenario:

*“The most memorable day I've ever had was here at XXX hotel; it was a holiday(x-mass) and was a tough day because there were so many people (customers). In the meantime, one of the customers shouted aloud at me for I did not bring the food he ordered me on time. Later, when I brought him the food, he threw it on my face; I was shocked in front of such large gatherings of people. I wish I would have died then. Instead of doing that, it would have been better had he beaten me. I lost control of myself; I was wondering why I'm doing this job.”(Participant No, Age, Store Name) e.g. (P1,24 years, Hotel)*

**Customers’ behavior and the Context**: the incidence of workplace violence against waitresses has been found to vary depending on customers’ behavior during and after drinking alcohol. According to the research participants, workplace violence increases during the night time in which customers get drunk. Week-ends have also been reported to be the days where waitresses are more exposed to the violence since many people, especially employees become free to enjoy, and often drink alcohol. Furthermore, exposure to workplace violence against women is also influenced by the specific setting in which the waitresses work. We found that waitresses working in grocery stores/bars are relatively more exposed to workplace violence than those working in restaurants and cafeterias.

Data revealed that though waitresses rarely experience violence from female customers, the most common perpetrators involved in inflicting violence against the victims are males. In addition, differences between male and female customers based on the types of violence they infiltrate to the waitresses have also been noted. In this regard, whereas waitresses mostly become targets of verbal violence from their same-sex customer counterparts, male customers are reported to engage in all types of violence, including physical violence and sexual abuse. Moreover, we found adults and relatively young customers as the most common

perpetrators of violence against the waitresses, while elderlies were reported to be least involved as far as the age of the perpetrators is concerned.

## Coping with workplace violence

Data reveal that waitresses adopt different mechanisms of surviving workplace violence. Ignorance has been reported to be the most commonly practiced type of coping strategy against workplace violence. Nevertheless, when waitresses choose to ignore responding to abusive circumstances, it is done after rational calculation of other alternative way outs. Interviewees disclosed that ignorance is motivated by fear of further consequences: *“I will speak nothing, especially to the customer. I might be upset, but I usually choose to immediately leave the area because I know that If I respond countering the deeds of the customer, the worst happens to me to the extent of perhaps losing my job.”* The sense of job insecurity observed among most waitresses is the result of the absence of formal procedures before and during employment. Many interviewees disclosed that both their first contact as well as later attachment to their current workplace has taken place through informal networks. Most have reported that they do not have signed contractual agreement specifying the rights and duties of both the employee and the employer and received no letter that clearly stipulates the roles and responsibilities (job description).

The following quote from an interviewee working in ……. Store demonstrates this reality: “*After completing my 10^th^ grade education, I was just sitting in the house and serving my family because I didn’t get a passing grade to continue preparatory education. In the meantime, I asked my friend who used to work in a cafeteria as a waitress to find me a vacant position. She then succeeded in finding it and told me to report to the owner. I was asked to bring a bail. Then, I called my relative who came and signed on a paper to guarantee accountability. It was in this manner that I started working. Except that, there is nothing I singed and received.”*

While most research participants prefer to pay no attention to quarrelsome circumstances in the workplace, they resort to reporting to the owners/supervisors when ignorance itself couldn’t end the process. We also found that waitresses’ decision to report to the owner/supervisor heavily relies on their trust that the owner is sympathetic and able to impartially understand their cause: *“…I was very angry and told the owner everything. Fortunately, the owner and the customer know each other. Then, the owner went and talked to him about what happened. Consequently, the customer paid the bill and finally left. Encountering to such violent customers is a common aspect of our daily life in this work. But, we inform to the boss when the insult becomes worse and she warns them not to come any more if they are about to abuse us like that. She will also tell us not to receive orders from such rude customers anymore.”*

Contrariwise, waitresses who are aware that the owner/supervisor is not dependable often resort to reporting to the police or opt to shout to the guards of the hotel. Mentioning that there are supervisors/owners that care about the safety of their employees' interviewees disclosed that some are less sympathetic that they take for granted violence against waitresses even when it is undertaken in their presence.

In response to facing frequent incidences of violence at a particular workplace and when other previously discussed avenues are proved not helpful, changing a workplace becomes another way some waitresses respond to workplace violence. Interviewees believe that working in relatively bigger hotels is less risky compared to smaller hotels, grocery stores, and cafeterias. A response of Liya, for instance, demonstrate the views held among waitresses about the less violent nature of customers to be served in the more prestigious hotels: “*As a way out, I have now decided not to work in small hotels; I will search for possible opportunities of working in bigger hotels because I think the customers to be served there are well educated and respectful. If I continue facing the same situation there, I change to a new field of work in the future.”* Furthermore, we found that some waitresses are study*i*ng in the extension modalities at private colleges in the area parallel to working as a waitress. Such waitresses unveiled their plan to quit the present job and their aspiration to be employed with the profession of their college trainings.

Data reveal that research participants vary depending on whether they have been informed ahead and hence, expecting the possibility of workplace violence before joining the sector. It is also found that such variations have influenced the extent to which waitresses differently felt the experience of violence and the way they respond to it. Whereas some interviewees disclosed that they were conscious of and expected the likelihood of facing workplace violence, they tend to assume the violence as the normal aspects of the work and are relatively less aggressive in reacting to the incidences. For instance, Alemitu disclosed:

*“obviously, I had the awareness; it was my cousin who suggested me to do this job. Because she was a waitress I used to hear from her when she was talking about the heavy workload and her ample experiences of diverse workplace violence. I finally found out that what I have faced and still facing is the same to her. But I already convinced myself before beginning the job. Even though I became angry, cried a lot, and felt lonely but I do not give up because I hope tomorrow will be fine.”*

Others have reported experiences of shocks while encountering the situation being under circumstances of lacking prior knowledge. For instance, Bekelech’s story demonstrates that the shock is temporary and some of such waitresses would prefer to remain in the work withstanding the shocks:

“*I had no idea about the job before joining it. I came into it all of a sudden following my failure to get a passing mark in 10^th^ grade. I was unemployed for a long time, and didn't think I would do this job; I thought I would start my own business. Subsequently, I joined the work just considering it as a temporary solution. Even if I frequently face different maltreatments, I try to convince myself that this time will pass and good days will come and persuaded myself to be patient.”*

## Discussion

Compared to their men counterparts, women are obliged to take on vulnerable forms of employment that fail to protect their basic labor rights (UNDP, 2018). Lippel (2018) argue that workplace sexual violence, and sexual harassment in particular, have received attention in the literature and in regulatory strategies, but other types of violence to which women are disproportionally exposed either at work, because of the nature of their work, or while they travel

to and from work, are less often addressed through a gender lens. According to EU-OSHA (2013), the level of exposure to workplace violence is especially high for those women who work within the agriculture, the hotels, restaurants and catering, the transport as well as the manufacturing sectors. It was in this context that the present research was undertaken. This study particularly focused on women working as waitresses in the various hotels, restaurants, grocery stores, and cafeterias of Wolaita Sodo town, southern Ethiopia, and explored the …………………………………………………………….Accordingly, the results of the study have shown that the incidence of workplace violence against waitresses in the study area is high. This result is consistent to Yemane (2004) who found that Ethiopia has one of the highest prevalence of sexual, emotional, and physical violence against women in the work place. According to Abera, et al. (2016), there is a high level of workplace injuries that often leads to an extended loss of productive working days in Ethiopia, where occupational safety and health services are inadequately organized. Moreover, a study of workplace violence among Turkish employees of various sectors undertaken by Akinci and Bilgel (2011) found that nearly half of the workers reported that they had been subjected to a violent event at their workplaces. According to Canadian Center for Occupational Health and Safety (2021) working with customers or the public is among the work factors that put people at increased risk of workplace violence and sellers of alcohol are among the occupational groups that tend to be at risk from workplace violence. Women are at particular risk of violence, harassment and bullying both in and outside the workplace (TUC, 2017; ILO, 2010).

The most commonly reported type of workplace violence in the present study is verbal abuse, mostly manifested in insisting the victims to provide their phone number to the male customers. Furthermore, waitresses are also found to be exposed to other forms of verbal abuses including insulting and humiliation, bulldozing/scaremongering and intimidation. In addition to the verbal abuse, incidents of physical violence have also been reported where slapping on the face, beatings, touching on sexual or private parts, spitting, twitting a hand, and simple physical assaults are commonly experienced by the waitresses. The finding is consistent to Akinci and Bilgel (2011) who found that the most common type of violence was verbal abuse, followed by bullying, physical assault and sexual harassment. A study of women’s health working in the mining sector by Botha (2015) revealed that women are still exploited and sexually harassed in the mining industry. Incidents taking place daily vary from whistling; name calling; use of vulgar or derogatory language; display of body parts; physical contact, ranging from touching to sexual assault and rape; to the exchange of sexual favors for promotion. Melak (2012), found a high prevalence of violence against female university students the rate of which ranges from the most frequent form, i.e., verbal harassment in the form of insult and catcall, to complete or attempted rapes which are the rare type of sexual violence against female students.

While we found high incidences of workplace violence against women in the study area, research participants also disclosed that the level of exposure to the violence varies across various circumstances, including working in large and small towns, the situation of the owners/supervisors, public’s insight of the position, waitresses’ ability to speak the local

language, the role of the waitresses, customers’ behavior and the specific context in which waitresses work. Workplace violence against women is rooted in perpetrator’s sense of power, control, and entitlement, gender-based stereotypes, perpetrator’s use of alcohol **a**nd other drugs, and victim’s blaming ideas, such as the belief that a victim somehow “asked for it” by the way s/he behaves, dresses or lives (Rutgers (2021). According to ILO (2011), risk factors for victims include their age, experience, being female, and attitudes and expectations (perceived vulnerability). Workplace risk factors are comprised of the working environment such as organizational setting and managerial style, as well as the workplace culture and external environment. Task-related risk factors include working alone, with the public and/or people in distress, and in educational settings. The chief causes for the exposure of females to different forms of violence are legal and structural, such as the absence of sound policy on girls in general and gender-based violence in particular. In addition, the absence of gender sensitivity in the legislations of organizations and the absence of a clear mandate and authority for the existing gender focal point institution is also another serious challenge (Melak, 2012). The existence of relevant national legislative frameworks on gender-based violence as well as a workplace-level policy and implementation strategy are also important contextual factors (ILO, 2011). Concerns of workers’ occupational health and safety have been included in Proclamation No. 1/1995 of the constitution of the Federal Democratic Republic of Ethiopia. Article 42 (2), for instance, provides that workers have the right to reasonable limitation of working hours, to rest, leisure, to periodic leaves with pay, to remuneration for public holidays as well as healthy and safe work environment.

Findings of the present study reveal that waitresses adopt different mechanisms of surviving workplace violence. Ignorance has been reported to be the most commonly practiced type of coping strategy against workplace violence, followed by reporting to the owners/supervisors, and changing a workplace. The result is once again consistent to Akinci and Bilgel (2011) who found that victims preferred to ignore or deny the violent act instead of fighting against it. According to their study, the most frequently reported way victims react to workplace violence was talking to colleagues about the violent act, followed by talking with family and friends and warning the perpetrator, “doing nothing,” “pretending nothing had happened”, and “getting help from the police.” Fredrickson (2014) found that the main reasons that employees do not report workplace violence include fear of their supervisor’s reaction, lack of company procedures/policies, lack of training, becoming the ‘office snitch’, and fear of retaliation. According to the finding of the author, employees afraid to report potential workplace violence to their supervisor because they think the supervisor will over-react or think they are unable to handle their own job/responsibilities.

Lack of information about women’s rights, limited access to legal services, insensitivity of law enforcement bodies, and the generally poor status of women in the society are some of the main reasons that accounted for the continuation of violence against women (Yemane, 2004). Above all, workplace violence can be considered as a reflection of the general and increasing

phenomenon of violence in many areas of social life which has to be broadly viewed at the level of the whole society (Ali & Ali, 2014).

## Conclusion

The results of the research indicated that waitresses are highly exposed to workplace violence both on account of their occupation of presumably a ‘less prestigious position’ and their sex. While waitresses are generally exposed to all forms of violence including spitting, throwing objects, simple physical assault, touching on private parts, and intimidation, verbal abuse and emotional exploitations are found to be the most experienced ones. Although waitresses rarely experience violence from female customers, the most common perpetrators involved in the violence were males. Above all, whereas the vulnerability of waitresses to workplace violence has been noted, level of exposure to the violence varies depending on the personal qualities of the owners/supervisors, the nature of the workplace itself and the socio-economic status of the customers served in those workplaces, waitresses’ ability to speak the local language, peoples’ perception of the position and responsibility of the waitresses, the manner in which the waitresses serve the customers, and the particular context in which waitresses work. Ignorance, mostly motivated by fear of losing one’s job, has been the common way waitresses respond to the violence in addition to …………………… ……………….. ………………… . The study implies the vulnerability state of waitresses partly due to lack of adequate awareness regarding the rights and obligations expected of an employee when working in such organizations. This is again exacerbated by the lack of formal employment procedures in such organizations. These circumstances have contributed to waitresses’ lack of job security and the fear of other possible further consequences when deciding to react to violence in the way it safeguards their rights. Furthermore, the wrong perception of the public about the position of waitress and the absence of concern from the side of the owners about workplace violence are also other aggravating factors. Hence, awareness creation and supervisory activities are expected from the media, workers’ and social security office of the government, police and other concerned bodies.

## **References**

Ali R., & Ali, M. (2014). A Review of the Causes of Workplace Violence and its Preventive Measures - The Management Perspective. Journal of Social Science Research, Vol. 4(3), 604-607.

Abera Kumie, Tadesse Amera, Kiros Berhane, Samet, J., Hundal, N., Fitsum G/Michael, Gilliland, F. (2016). *Ethiop. J. Health Dev.* 30(Special Issue):17-27

Action aid. (2012). The theory of change for tackling violence against women and girls. Gender and development network

Akinci, S. and Bilgel, N. (2011). Workplace Violence: A study of Turkish Workers. *International Journal of Occupational Safety and Ergonomics* (*JOSE*) 2011, Vol. 17, No. 4, 385–402

Alam, A. (2010). Impact of gender discrimination on gender development and poverty alleviation. *Sarhad J. Agric. 27(2): 329-339*

Amnesty International. (2004). *It’s in our hands: Stop violence against women*. Osney Mead, Oxford, United Kingdom: Alden Press.

Amoako, J. (2019) "Women's occupational health and safety in the informal economy: Maternal market traders in Accra, Ghana". Dissertations and Theses @ UNI. 951.

Banerjee, B. (2009). Physical hazards in employment and pregnancy outcome. *Indian Journal of Community, 34*(2), 89-93. doi: 10.4103/0970-0218.51224

Botha, D. (2016). Women in mining still exploited and sexually harassed. *SA Journal of Human Resource Management/SA Tydskrif vir Menslikehulpbronbestuur*, *14*(1), a753. [http://dx.doi.](http://dx.doi/) org/10.4102/sajhrm. v14i1.753

Canadian Center for Occupational Health and Safety (CCOHS). (2021). Violence and Harassment	in	the	Workplace.	Retrieved	online	from <https://www.ccohs.ca/oshanswers/psychosocial/violence.html>

Coyle, D., Shrestha, R., & Jung, C. (2014). Women’s insecurities and the workplace in Nepal: A study from Banke and Bara districts. Forum for Women, Law and Development, Informal Sector Service Center, Institute of Human Rights Communication Nepal, International Alert, National Business Initiative, Saferworld,

Dawit Seblework. (2006). Occupational Safety and Health Profile for Ethiopia. ILO & Ministry of Labor and Social Affairs

EU-OSHA. (2013). Risks and Trends in the Safety and Health of Women at Work. European Risk Observatory: A summary of an Agency report

EU-OSHA, (2014). New Risks and Trends in the Safety and Health of Women at Work. European Agency for Safety and Health at Work Office for Official Publications of the European Communities, Luxembourg.

Federal Negarith Gazeth. (1995). Constitution of the Federal Democratic Republic of Ethiopia, Proclamation no. 1/1995

Fredrickson, C. (2014). Seven Reasons Employees Don’t Report Workplace Violence, Retrieved online from <https://www.businessknowhow.com/manage/reportviolence.htm>

Gatimu, C. (2015). An Analysis of Sexual and Gender Based Violence (SGBV) Interventions in South Sudan. OCCASIONAL PAPER, SERIES 6, No 3, International Peace Support Training Center Nairobi, Kenya

ILO, (2010). Women Workers and Gender Issues on Occupational Safety and Health.

International Labour Organization, Geneva.

ILO. (2011). Gender-based violence in the world of work: overview and selected annotated bibliography, International Labor Office. – Geneva: ILO, 1 v.

Kane, P. (nd). Introduction: Women and Work in a Changing Environment. Issues and policy paper prepared for the Global Commission on Women's Health. The Office for Gender and Health, the University of Melbourne

Koh, D. & Jeyaratnam, J. (2001). Work and Health: Textbook of Occupational Medicine Practice. Koh H, Seng, C.K. Jeyaratnam J. (editors). 2^nd^edition. Singapore: World Scientific.

Labor and Employment Law. (2003). PROCLAMATION No. 377/2003 LABOUR PROCLAMATION,	retrieved	from file:///D:/women%20waitress/Ethiopia/PROCLAMATION%20No.%20377_2003%20LA BOUR%20PROCLAMATION%20-%20Ethiopian%20Legal%20Brief.html

Lippel, K. (2018). Conceptualizing Violence at Work Through A Gender Lens: Regulation and Strategies for Prevention and Redress, *University of Oxford Human Rights Hub Journal, Vol. 1, 143-166*

Lu, J. L. (2011). Occupational Health and Safety of Women Workers: Viewed in the Light of Labor Regulations. *Journal of International Women's Studies*, *12(1), 68-78.*

Melak Mengistab. (2012). Gender Based Violence against Female University Students in Ethiopia: The Case of Bahir Dar University. Unpublished Thesis Submitted to the Graduate School of Addis Ababa University in Partial Fulfillment of the Requirement of the Degree of Master of Arts in Human Rights

Morrison, A., Ellsberg, M., Bott, S. (2004). Addressing Gender-Based Violence in the Latin American and Caribbean Region: A Critical Review of Interventions. World Bank Policy Research Working Paper 3438, October 2004

Mukanangana, F., Moyo, S., Zvoushe, A., Rusinga, O*. (*2014)*.* Gender Based Violence and its Effects on Women’s Reproductive Health: The Case of Hatcliffe, Harare, Zimbabwe *Afr J Reprod Health, Vol. 18[1]: 110-122*

Pedro, S. (2013). Communication Strategies for Preventing Violence against Women: Case Study of Timor-Leste. *Universidade Autónoma de Lisboa, Portugal, Estudos em Comunicação* nº *14, 1-38*

Rajbhandari R, Subedi S, Kaphle HP. (2015). Workplace violence against health workers: a cross-sectional study from Baglung district, Nepal. Int J Health Sci Res. Vol. 5(2):274- 281.

Rutgers. (2021). Contributing factors- Violent prevention and Victim Assistance. Rutgers, the state university of new Jersy. An Equal opportunity, Affirmative Action

Subedi, S., Hamal, M., Hari, P. (2013). Sexual Harassment in the Hospital: Are Nurses Safe?

*International Journal of Health Sciences & Research, Vol.3 (6), 41-47*

Tawiah, K. & Mensah, J. (2016). Occupational Health and Safety and Organizational Commitment: Evidence from the Ghanaian Mining Industry Saf Health Work; 7:225e230 Tsegahun Tessema. (2008). The Status of Gender Based Violence and Related Services in Four Woredas (Woredas surrounding Bahir Dar town, Burayu woreda, Bako woreda and

Gulele Sub-city of Addis Ababa), CARE Ethiopia.

TUC. (2017). Gender in occupational safety and health: A TUC guide for trade union activists (with gender checklist)

UNDP. (2018). ETHIOPIA | National Human Development Report: Industrialization with a Human Face, United Nations Development Program, Ethiopia country office

WHO (World Health Organization). (2001). Occupational Health: A manual for primary health care workers, World Health Organization, Regional Office for the Eastern Mediterranean

WHO	(World	Health	Organization).	(2018b).	Occupational	health.	Retrieved	from <http://www.who.int/topics/occupational_health/en/>

WHO (World Health Organization). (2004a,). Gender, health and work. *Gender and Health.*

Retrieved from [http://www.who.int/gender/other_health/Gender,HealthandWorklast.pdf](http://www.who.int/gender/other_health/Gender%2CHealthandWorklast.pdf) Yemane Berhane. (2004). Ending Domestic Violence against Women in Ethiopia. *Ethiopian*

*Journal of Health Development, DOI: 10.4314/ejhd.v18i3.9951*
