# Supplementary material for: Victimizations and surviving of workplace violence against waitresses in southern Ethiopia
Source: PLoS One. 2021 Dec 31;16(12):e0261883. doi: 10.1371/journal.pone.0261883 (PMC8719772; doi:10.1371/journal.pone.0261883)
Supplement: Attachment — Submitted filename: Point-by-point response.docx [file pone.0261883.s003.docx]

**Point-by-point Response**

Dear Dr. Samantha, we would like to express our sincere gratitude to you for your editorial services and your help in the process of enabling the peer review process to be undertaken within a reasonable time frame. We are also thankful to the reviewers that have devoted their time for reviewing the material and contributed their share for improving the quality of the article. We wholeheartedly believe that the comments and suggestions provided herewith are highly helpful in terms of producing an interesting paper.

Dear all, below is a table containing the comments of the reviewers (and the editor) and a point-by-point response of the authors. We believe that you will find satisfying responses for all the concerns you raised in the paper. If there are further issues that need to be addressed, please do not hesitate to contact us any time. Due to our ambition to get the article published very soon, the corresponding author will be staying 7/24 online attending your emails.

Dear editor, in an attempt to ease the editorial process and thereby fasten the publication, we have revised the reference section and changed the style of the in-text citation (in superscript form) according to the journal’s style. Above all, the data collection instrument has also been attached at the end of this page.

Thank you very much once again!

| source of Comment | Details of Comments | Authors’ Responses |
| --- | --- | --- |
| Reviewer #1 | - Introduction –-This section looks good, but it has plenty of information. It needs to be shortened by condensing the information that are related to workplace violence and women, as well as by removing the duplication (in information) and other theoretical description that are not specific with the objectives of the study. For example, this paper only focuses on the status of workplace violence and does not have anything related to policy/legislative aspects. So, it is better to remove that. | - Corrected accordingly! - In addition to correcting all the recommended points, we have also revised the whole introductory section, to the extent of paraphrasing it. |
|  | - Materials and methods –Overall good. However, it needs a bit more clarification on the selection of participants (e.g., the number of participants selected from each store). The method of data analysis needs more detailed description on how the data were analyzed. Ethics related aspects (privacy, confidentiality, ethical approval, etc.) are missing. | - Corrected accordingly! - The ethics statement is provided as a separate section, data analysis method clarified, sampling again clarified. |
|  | - Results –This section is very good except the style of presenting the information (layout) and some grammatical errors. Structure or layout needs to be rearranged, such as change of paragraph while mentioning the statement of participants. Some statements given by participants are in present tense, which needs to be corrected. There are names in few statements which does not seems to be ethical. Participant's name must be removed and de-identified, but it does need the other characteristics of participants such as their age and working store. E.g. P12, 26 years, Cafeteria | - corrected accordingly! |
|  | - The starting of the discussion section does not seem to be appropriate. It should be started with the background of the results/objectives. Do not repeat the whole results but briefly mention your results and discuss it with other. While discussing, focus on why and how of your findings as well as other study's findings. At the end of discussion section, write few sentences on how your findings are significant or different than other studies and its implication in Ethiopian context. | - Corrected according to the suggestion. - As far as inclusion of just major findings is concerned in the discussion, we have made at most effort of not missing interesting findings while at the same trying not to go to unnecessary details. |
|  | - Referencing in the text as well as in the bibliography section does not match the PLOS guidelines. It is meant to be in Vancouver style, but this manuscript adopted Author-Date/Harvard method. It needs major revision. Moreover, references need to be cross checked between the text and the bibliography section. | - corrected accordingly |
| Reviewer #2 | - It would be good to have a theory for the study | - Dear reviewer, thank you for your insightful suggestion. we are afraid that inclusion of additional literatures would make the introductory section unnecessarily too much. Of course, it could have been done by reducing some portions from the introductory section. But we still believe that sufficient background and justification have been provided in the section. We hope you will understand and share our concern. |
|  | - The sampling framework needs to be clear. For example, Woliata sodo sounds like a small town and it would be important to mention about how many groceries, restaurants etc that are there, so that we can have a feel of the representation. | - Dear reviewer, it is true that Wolaiata Sodo is relatively a small town-actually not that too small. The problem is not from the size of the town, rather it is about the availability of data from the concerned gov’t office. When asked, what the officers give us is just an estimation arrived without data. - In fact, we have made certain clarifications to the sampling section |
|  | - “We have purposefully selected equal number of participants representing the group of waitresses working in the restaurant, cafeteria, hotel, and grocery store”- this statement can be challenged because some of the places may have fewer workers than others, which means that some organization may end up having more participants than others. Could there be some restaurants, cafeteria having fewer women employees than the selected ten? | - we have revised the section accordingly! - thank you |
|  | - Another question is the criteria used to select the ten from each organization? | - revised |
|  | - Whereas it is fair to say that “data saturation was attained after sixteen waitresses were interviewed”; one is left wondering about the representation of the 16---was there a good representation from the four sectors, that is, restaurant, cafeteria, hotel, and grocery? | - the section has been revised accordingly |
|  | - “Sex and willingness to participate in the study were the major inclusion criteria we used”; again, the question is whether each person was approached and were asked whether they were willing to participate? | - Willingness of waitresses to participate in the study was asked after inclusion at the initial sampling stage-40. Dear reviewer, the 16 were the ones who finally given their full consent to participate. |
|  | - Ethical issues have not been addressed in this very sensitive topic | - now addressed |
|  | - Research approvals have not been mentioned | - now considered |
|  | - Results & Discussions: they are very interesting, but it would have been interesting to hear voices from the different industries. For example, from the analysis, what kind of violence is mainly experienced by those in the cafeteria industry/ or to describe the direct quote verses (e.g. said so and so who works in the grocery store. Further, the language needs to be reworked on in this section. For example, is the coping strategy ignorance or ignoring – the two terms are different, and it feels like they have been used interchangeably. Direct quotes need to be restructured so that they make sense to the readers. Some of the paragraphs are too long especially under the discussion heading. | - revised |
|  | - In the discussion section, the key findings need to be captured as the existing literature/ empirical findings are discussed. | - revised |
|  | - Make the conclusion strong so that we hear the gap that existed and how it has been met through this study. | - Considered. The gaps and how they were addressed, the difference with previous studies, and the contribution of the study all are discussed in the discussion in addition to the conclusion. |

In-depth Interview guiding questions

1. How prevalent is gender-based violence in your work place?
   1.1. Have you ever faced gender-based violence now or before while working in similar work places?
   1.2. Would you please tell me some of the violence types that you faced?
   1.3. ………..*also ask her to specify where, when (what time), by whom ( whether she has faced it from the side of customer/client, employer/owner, owner’s family/relative, stranger, etc)*
   1.4. *……also specify if she faced the violence on account of her gender, ethnicity, religion, social class, or any other stratifying variable?*
   1.5. Have you ever faced violence or discrimination on the basis of your ethnicity, religious affiliation, or belongingness to a social group that is perceived to as a different/minority in the area?
2. How did you react or respond to the violence you have faced?
   2.1. What did you do after facing the violence or discrimination in your work place?
   2.2. Have you ever reported to the police, managers/supervisors, [body] guards, the owner, etc……or you just kept silent?
   2.3. ……….and why did you do that ……or not did so?
   2.4. If you have ever reported, then how did they/it respond to your complaints?
   2.5. …………..What is the most common thing you do whenever you face such violence?
3. What physical, psychological, and social challenges have you faced because of the violence?

……… *social exclusion, physical damage, illness or sickness, fear or feelings of intimidation, depression, anxiety, loneliness, feelings of helplessness, feelings of worthlessness, etc*

4. What was your expectation regarding the possibility of being exposed to gender-based violence in such work places?
   4.1. Have you ever expected or were you expecting to be exposed for such workplace violence when you decide to work here?
   4.2. ….if yes, then, have you convinced yourself to accept victimizations?
   4.3. …….what strategies have you planned to cope-up with the situation?

| No (code) | Variable | Categories | Frequency |
| --- | --- | --- | --- |
|  | Age |  |  |
|  | Marital status |  |  |
|  | Education |  |  |
|  | Years in the work |  |  |
