# Supplementary material for: Victimizations and surviving of workplace violence against waitresses in southern Ethiopia
Source: PLoS One. 2021 Dec 31;16(12):e0261883. doi: 10.1371/journal.pone.0261883 (PMC8719772; doi:10.1371/journal.pone.0261883)
Supplement: Attachment — Submitted filename: Response to reviewrs & the editor - 2.docx [file pone.0261883.s005.docx]

**Responses to Reviewers and the Editor**

Dear Dr. Winter, we would like to express our sincere gratitude to you for your editorial services and your help in the process of enabling the peer review process to be undertaken within a reasonable time frame. We are also thankful to the reviewers that have devoted their time for reviewing the material and contributed their share for improving the quality of the article. We wholeheartedly believe that the comments and suggestions provided herewith are highly helpful in terms of producing an interesting paper.

Dear all, below is a table containing the comments of the reviewers (and the editor) and a point-by-point response of the authors. We believe that you will find satisfying responses for all the concerns you raised in the paper. If there are further issues that need to be addressed, please do not hesitate to contact us any time.

Dear editor, in an attempt to ease the editorial process and thereby fasten the publication, we have revised the reference section and changed the style of the in-text citation (in superscript form) according to the journal’s style.

Thank you very much once again!

| source of Comment | Details of Comments | Authors’ Responses |
| --- | --- | --- |
| Reviewer #1 | - Although most of the comments have been addressed, the manuscript still needs revision especially in sentence structure, grammar, and format/style. I highly recommend authors to take referencing pattern/citation seriously and revise it. | - Revised |
|  | - In abstract (very initial pages, not the text one), correct the capitalization on keywords. | - Corrected |
|  | - There are still some issues in referencing. For example, in the introduction section, there are few sentences that says According to [8], various sectors undertaken by [30], health working in the mining sector by [26] revealed. This is not the way how you do citation. You can write the author's name in this type of sentences (although it is in Vancouver) and mention the references in number at the end of the sentence OR write it as a finding and keep the reference at the end. For example ----- Instead of writing "Moreover, [27] found high level of morbidity in the form of headache, body ache, problems with vision, cough and breathlessness in Brick Kiln and Construction industries of India" simply write "high level of morbidity in the form of headache, body ache, problems with vision, cough and breathlessness in Brick Kiln and Construction industries of India [27]. | - Revised |
|  | - No need to put reference in the first sentence of 'research design' | - Removed |
|  | - Keep an eye on the capitalization of middle words. Example – "Selection of Research participants" Under the section "selection of research participants", there are notable errors in grammar. Methodology should be always in past tense, not in present tense. Same types of errors still exist in 'Findings'. Example – "Data reveal that waitresses adopt", "data reveal that waitresses often contribute to", "Data revealed that though waitresses rarely experience"…… and so on. | - Revised |
|  | - In findings section, it's good to break the paragraph rather than placing chunk of information and quotations in one paragraph. For example, see the sub-section "the situation of the owners/manager". | - Corrected |
| Reviewer #2 | - The author(s) have improved on the document and critically looked into the issues that were raised during the first review. The issues has been presented well and I hope the findings will get to the necessary policy makers or influencers of the same. | - Thank you |
| Editors’ comments | - The authors changed the in-text referencing style from one which used author names and dates such as APA (e.g., Adams, 2019) to a numerical referencing style; however, when they did this, all of the references to specific studies in the introduction and throughout the discussion became inappropriately referenced, (e.g. according to [8],...). These need to be corrected. | - Revised |
|  | - Additional proofreading for typographical, editorial, and grammatical errors is needed. | - Revised |
